# Cilia are required for asymmetric *nodal* induction in the sea urchin embryo

**DOI:** 10.1186/s12861-016-0128-7

**Published:** 2016-08-23

**Authors:** Matthias Tisler, Franziska Wetzel, Sabrina Mantino, Stanislav Kremnyov, Thomas Thumberger, Axel Schweickert, Martin Blum, Philipp Vick

**Affiliations:** 1 University of Hohenheim, Institute of Zoology, 70593 Stuttgart, Germany; 2 Department of Embryology, Lomonosov Moscow State University, Moscow, Russia; 3 Present Address: Centre for Organismal Studies, Im Neuenheimer Feld 230, Heidelberg University, 69120 Heidelberg, Germany

**Keywords:** Sea urchin, Deuterostomes, Left-right asymmetry, Symmetry breakage, Nodal, Cilia, Left-right organizer

## Abstract

**Background:**

Left-right (LR) organ asymmetries are a common feature of metazoan animals. In many cases, laterality is established by a conserved asymmetric Nodal signaling cascade during embryogenesis. In most vertebrates, asymmetric *nodal* induction results from a cilia-driven leftward fluid flow at the left-right organizer (LRO), a ciliated epithelium present during gastrula/neurula stages. Conservation of LRO and flow beyond the vertebrates has not been reported yet.

**Results:**

Here we study sea urchin embryos, which use *nodal* to establish larval LR asymmetry as well. Cilia were found in the archenteron of embryos undergoing gastrulation. Expression of *foxj1* and *dnah9* suggested that archenteron cilia were motile. Cilia were polarized to the posterior pole of cells, a prerequisite of directed flow. High-speed videography revealed rotating cilia in the archenteron slightly before asymmetric *nodal* induction. Removal of cilia through brief high salt treatments resulted in aberrant patterns of *nodal* expression. Our data demonstrate that cilia - like in vertebrates - are required for asymmetric *nodal* induction in sea urchin embryos.

**Conclusions:**

Based on these results we argue that the anterior archenteron represents a *bona fide* LRO and propose that cilia-based symmetry breakage is a synapomorphy of the deuterostomes.

**Electronic supplementary material:**

The online version of this article (doi:10.1186/s12861-016-0128-7) contains supplementary material, which is available to authorized users.

## Background

Vertebrates possess pronounced visceral asymmetries along their left-right (LR) body axis, although they belong to the large phylogenetic group of the Bilateria, which refers to their bilaterally symmetric outer appearance [1]. Most organs are positioned in a characteristic way in the thoracic and abdominal cavities. In all vertebrate species examined so far, these asymmetries are under the control of the Nodal signaling cascade, which is only activated in the left lateral plate mesoderm before the first appearance of anatomical asymmetries [2]. The secreted transforming growth factor beta (TGFβ) Nodal binds to its receptor which results in the activation of its own transcription and that of its negative feedback inhibitor *lefty* (*left-right determination factor*), another secreted TGFβ superfamily member. Lefty antagonizes Nodal signaling, providing its temporal and spatial control. Additionally, the paired-like homeodomain transcription factor *pitx2* is induced downstream of Nodal and mediates, through less well-known target gene activation, the setup of asymmetric organ morphogenesis.

The event activating this highly conserved developmental program is referred to as symmetry breakage. Even though variations of the common theme may exist, an ancestral mode of vertebrate symmetry breaking has emerged over the past few years: at the heart of this mechanism acts an extracellular leftward fluid flow, generated by a transient ciliated epithelium, the so-called left-right organizer (LRO) [3–5]. The vertebrate LRO (known as Kupffer’s vesicle in fish, gastrocoel roof plate (GRP) in amphibians, and posterior notochord or ventral node in mammals) represents a field of mono-ciliated cells at the posterior end of the forming notochord, flanked by endodermal archenteron cells [6–8]. This unique tissue, which develops during early neurula stages, consists of superficially located mesendodermal cells which are transiently placed in the primitive gut or archenteron, where they function as the LRO. The cells of the LRO subsequently leave the epithelium to ingress into deeper mesodermal structures [7, 8]. The polarized attachment of cilia at the posterior cell surface, together with their clockwise rotational movement, create an asymmetric stimulus by the leftward acceleration of extracellular fluids, i.e. a leftward flow [9]. This setup has been functionally described in mammals (mouse, rabbit), amphibians (*Xenopus*) and teleost fish (medaka, zebrafish), and homologous tissues have been identified in salamanders (axolotl) and basal bony fish (white sturgeon). Interference with ciliary length, motility, polarization or flow function in general resulted in defects of the LR axis [10–15].

As a result of leftward flow, the Nodal inhibitor *dand5* becomes down-regulated on the left margin of the LRO, where it is co-expressed on both sides with *nodal* itself. Nodal thus becomes liberated on the left side to induce the asymmetric signaling cascade in the left lateral plate mesoderm [16–18]. *dand5* repression is induced through a flow-dependent intracellular calcium signal, which is mediated through the calcium channel pkd2 (polycystic kidney disease 2). Although ubiquitously expressed during these stages, pkd2 inhibition causes LR defects and prevents a unilateral calcium signal in mouse and fish LROs [5, 19–22].

Outside of the vertebrate lineage, LR asymmetries are common as well. For many deuterostome lineages, unilateral *nodal* expression has been described, which seems to be *the* common mediator of LR asymmetries in metazoan animals [3, 23–25]. Tunicates, the sister group of the vertebrates, as well as the cephalochordates, the most basal group of chordates, both express *nodal* asymmetrically on the left side [26, 27]. The latter seem to show an ancient state of this set-up, with many LRO targets activated similarly to vertebrates, such as *nodal*, *lefty*, *pitx2* and *dand5* [26, 28–31]. Accordingly, interfering with Nodal activity in the cephalochordate amphioxus also resulted in LR defects [32]. The existence of an LRO in amphioxus has been predicted, but not yet analyzed or functionally tested [3, 11]. Within the deuterostomes, the Ambulacraria, a chordate sister group which comprises the echinoderms, show unilateral *nodal* activation during late gastrula stages as well [33]. Although adult echinoderms took the path of reestablishing a radially pentameric body plan during evolution, their early embryo displays the typical bilaterally symmetrical embryonic development, which results in a pronounced LR asymmetry before metamorphosis [34]. As in other deuterostomes, *nodal* is the first asymmetrically activated gene, and is first found on one side in the developing archenteron tip [33, 35]. The functional requirement and a complex downstream gene regulatory network have been elucidated in detail in sea urchin embryos [36]. This asymmetric archenteron domain instructs a second symmetrical ectodermal *nodal* domain to switch towards this side as well [37]. This expression is thought to be on the *right*, as deduced from a ventral mouth opening [25]. We have previously argued that this difference can be resolved to a common evolutionary origin, if the ventral side of echinoderms is homologized with the dorsal side of chordates [11]. This reasoning is based on the expression of dorsal organizer genes in the oral ectoderm of the sea urchin larva, suggesting that the mouth - counterintuitively - opens on the dorsal side. Such an evolutionary mouth repositioning can be easily envisaged as a transitional process from echinoderm ancestors to vertebrates [38]. Indeed, oral organizer identity has been recently shown to be functionally conserved in the sea urchin *P. lividus* [39]. Thus, in this scenario, *left*-asymmetric *nodal* expression is a synapomorphy of the deuterostomes [11].

One major question, however, has remained unanswered: how does symmetry breakage upstream of asymmetric *nodal* induction occur in echinoderms? Do sea urchin embryos possess a LRO or an evolutionary functional precursor that induces asymmetric *nodal* expression? Do archenteron cells possess cilia and, if so, are these required to induce asymmetric *nodal* expression? Using descriptive and functional approaches we show that (1) archenteron cells in the sea urchin larva harbor monocilia; (2) archenteron cilia are polarized and motile; and (3) cilia are required for asymmetric *nodal* induction.

## Results

### Sea urchin archenteron cells harbor polarized monocilia

**Fig. 1:**
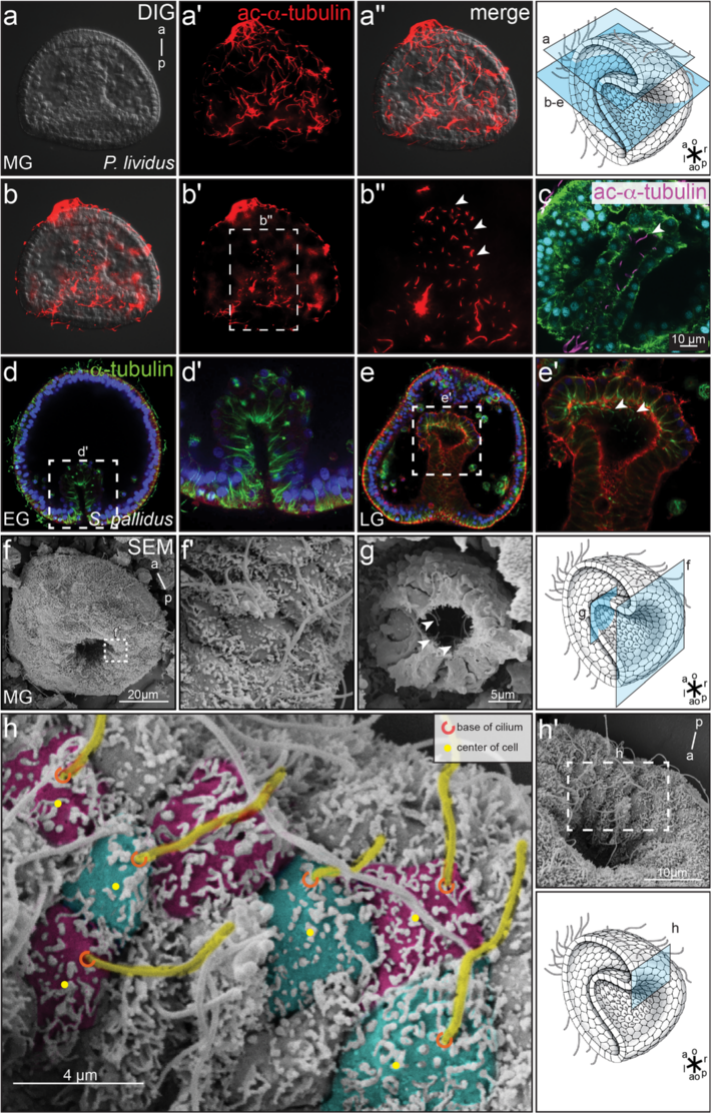
Polarized cilia at the sea urchin archenteron. **a**–**e** *P. lividus* (**a**–**c**) and *S. pallidus* embryos (**d**–**e’**) were analyzed by IF for the presence of cilia at the archenteron. Optical sections showed ectodermal (**a’**; **a”**) and archenteron cilia (**b’**–**c**) at mid gastrula stages. Late (**e**; **e’**) but not early *S. pallidus* gastrula stage embryos revealed cilia in the archenteron and at the archenteron tip (**d**, **d’**). Cilia were stained with an antibody against acetylated-α-tubulin (red, **a’**–**c**) or anti-α-tubulin (green, **d**–**e’**), nuclei were stained with DAPI (**d**–**e’**), and cell boundaries were visualized by phalloidin-green (**c**) or phalloidin-red (**d**–**e’**). **f**, **g** SEM analysis of *P. lividus* ectodermal and archenteron cilia. Fractured embryos allowed the visualization of monocilia on archenteron cells (**g**). Cilia are highlighted by an arrowhead (**f**–**h’**) Posterior polarization of cilia on cells which invaginated into the archenteron. Cilia are colored in yellow and individual cells alternating in green and purple. The ciliary base is marked by a red semicircle, the center of the cell is indicated by a yellow dot. Schematic drawings adapted from Blum et al. 2014 [3]

The defining feature of vertebrate LROs are polarized monocilia. As an entry point into studying sea urchin symmetry breakage, we investigated the presence of cilia on staged gastrula embryos by performing immunofluorescence (IF) with a well-characterized anti-acetylated α-tubulin antibody. In addition to the previously described long ectodermal cilia, optical sections of gastrula stage *Paracentrotus lividus* embryos revealed a population of shorter cilia within the developing archenteron (Fig. 1a–b”). Higher magnification showed that most cilia ranged from 4–6 μm in length, with the most anterior ones being longer, measuring up to 10 μm (Fig. 1c). The same basic characteristics of mesendodermal archenteron cilia were also found in a second sea urchin species, *Strongylocentrotus pallidus*, demonstrating the conserved presence of cilia in the primitive gut of sea urchin larvae. One slight variation was observed, namely that very early gastrula embryos of *Strongylocentrotus* apparently lacked archenteron cilia (Fig. 1d), which were, however, present at mid to late gastrula stages (Fig. 1e). In *P. lividus*, cilia were present already at earlier gastrula stages (Fig. 1b, and data not shown).

Scanning electron microscopy (SEM) of *P. lividus* was employed to further characterize archenteron cilia and to test a potential polarization of cilia along the animal-vegetal, and thus along the AP axis of the embryo (Fig. 1f–h). Embryos, which were broken perpendicularly to the animal-vegetal axis and thus allowed a high-power magnification view inside the archenteron, revealed monocilia of about 4 μm in length (Fig.1f, g). Archenteron cilia were clearly present already in mid gastrula stage embryos (Fig. 1f). Importantly, using the cilium-insertion point and the cell center as reference points, a clear posterior polarization of cilia was obvious already when cells were orienting towards the inside of the archenteron in animal/anterior direction (Fig. 1h). In summary, our descriptive analysis of the archenteron showed that cells harbored monocilia at a time point just prior to the asymmetric induction of *nodal*, suggesting a functional role homologous to that of vertebrate LROs.

### The sea urchin gastrula embryo expresses marker genes for motile cilia

**Fig. 2:**
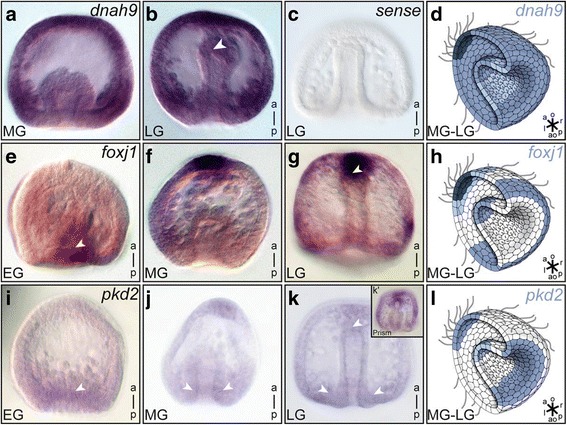
Motile cilia marker genes *dnah9* and *foxj1* are expressed throughout sea urchin gastrulation. Whole mount in situ hybridization of early (EG), mid (MG) and late (LG) gastrula stage *P. lividus* embryos, as well as prism stages (**k’**) for mRNA expression of *dnah9* (**a**–**d**), *foxj1* (**e**–**h**) and *pkd2* (**i**–**l**). Schematic representation of staining in mid-gastrula embryos is highlighted in drawings in (**d**, **h** and **l**). White arrowheads highlight vegetal blastopore and archenteron tip expression areas. Schematic drawings adapted from Blum et al. 2014 [3]

Next we analyzed the expression of marker genes indicative of motile cilia. To that end, sea urchin homologs of two marker genes for motile cilia, *dynein axonemal heavy chain 9* (*dnah9*) and *forkhead box protein J1* (*foxj1*) were cloned by RT-PCR and expression patterns during embryonic development were assessed by whole-mount in situ hybridization. In the ectoderm, *dnah9* was broadly expressed with intense signals in the apical tuft region. Localized mRNA expression was also found in the vegetal part of the gastrula mesendodermal tissue, followed by expression in the archenteron (Fig. 2a, b). Sense control probes were negative at all stages examined and for all genes analyzed in this study (Fig. 2c and data not shown). Analyses of *foxj1* mRNA expression revealed a similar pattern in the ectoderm and strong staining in the area of the developing apical tuft (Fig. 2e–g). At early gastrula stages, localized expression was found in the vegetal plate region (Fig. 2e), while in late gastrula stages, a mesodermal expression domain started to appear at the anterior tip of the archenteron (Fig. 2g). These expression patterns were indicative of a population of motile mesendodermal cilia in the archenteron and reminiscent of vertebrate LROs.

To investigate additional potentially conserved LRO genes, we cloned *P. lividus Bicaudal C homolog 1* (*bicc1*) [40], which is required for cilia polarization at the vertebrate LRO, and *pkd2*, a calcium channel required for sensing of the flow [22]. Expression of *bicc1* has been recently described in the sea urchin *Hemicentrotus pulcherrimus*, i.e. an unrelated genus. Our analysis in *P. lividus* confirmed the reported localization, i.e. strong expression of *bicc1* mRNA at the vegetal pole of early to late gastrula stage embryos, directly at the site of invagination (Additional file 1: Figure S1 and [41]). *P. lividus pkd2* mRNA (also termed *suPC2*) was expressed in a pattern reminiscent of *bicc1* in early to late gastrula stages, namely in vegetal cells of the early gastrula embryo (Fig. 2i–l). *pkd2* mRNA transcription was further activated in the apical tuft cells and at the archenteron tip of late gastrula to early prism stage embryos (Fig. 2i–k).

### Cilia in the archenteron are motile

In order to directly assess the potential motility of archenteron cilia in live embryos, we analyzed early to mid-gastrula stage embryos by high-speed videography. To highlight moving objects, movies were processed using a temporal difference imaging method (cf. Material and Methods). Additional file 2: Movie 1 demonstrates that the posteriorly polarized cilia of invaginating archenteron cells (cf. Fig. 1g) were indeed highly motile. Next we analyzed mid to late gastrula stage embryos, focusing on the lumen of the central part of the elongated archenteron. Again, the monocilia detected by SEM analysis (Fig. 1h) were motile, displaying a rotating pattern (Additional file 3: Movie 2). Attempts to visualize a possible effect of cilia motility, i.e. whether or not this resulted in directed movement of extracellular fluids, failed due to technical reasons, as we were not able to introduce fluorescent micro beads into the archenteron (not shown). Despite this shortcoming, our results strongly suggest that the sea urchin embryo harbors a vertebrate-type LRO, i.e. an archenteron epithelium with posteriorly polarized rotating monocilia, which expresses a set of characteristic marker genes, including *foxj1*, *dnah9*, *bicc1* and *pkd2*.

### Archenteron cilia are required for asymmetric *nodal* induction

As an alternative to visualizing cilia-driven fluid flow, we chose to directly test the function of cilia in asymmetric *nodal* induction. In a first set of experiments we used the pharmacological inhibitor Ciliobrevin D, which inhibits the ATPase activity of axonemal and cytoplasmic dynein motor proteins [42]. Treatment of early gastrula stage embryos with 50 μM Ciliobrevin D efficiently inhibited ciliary motility, as judged by direct microscopic observation of embryonic swimming behavior, which completely ceased within minutes (data not shown). As this treatment efficiently prevented gastrulation movements as well, we were not able to analyze later *nodal* expression (data not shown).

**Fig. 3:**
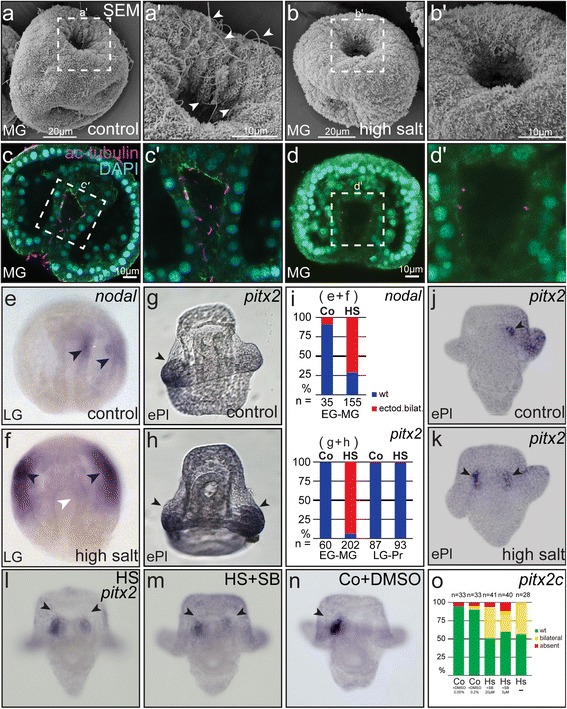
Deciliation impairs LR asymmetric *nodal* cascade induction. SEM (**a**, **b**) and IF (**c**, **d**) analyses of cilia in control untreated embryos (**a**, **c**) and specimens exposed to a 60–90 s osmotic shock (**b**, **d**). Note that cilia were almost completely absent following high salt treatment. Cilia in (**c**) and (**d**) were stained with an antibody against anti-acetylated-α-tubulin (purple), nuclei with DAPI, and cell boundaries visualized by phalloidin-green. **e**, **g** Unilateral induction of asymmetric *nodal* cascade genes in control embryos. **f**, **h** Bilateral ectodermal *nodal* and *pitx2* expression following deciliation of early to mid-gastrula stage embryos. Black arrowheads highlight expression in the archenteron and ectoderm; white arrowheads indicate lack of expression. **i** Quantification of expression pattern from (**e**–**h**). Unilateral *pitx2* expression in coelomic pouches of pluteus stage control specimen (**j**) and bilateral expression in deciliated embryos (**k**). Bilateral expression of *pitx2* after deciliation is independent of MAPK/p38 inhibition through SB203580 (**l**–**m**). **o** Quantification of expression patterns. Posterior/vegetal is to the top in (**a**, **b**) and to the bottom in (**c**–**g**)

Short of a specific inhibitor of axonemal dynein function, we decided to assess the role of cilia for symmetry breakage by removing cilia from the embryo. To that end, a brief osmotic shock with high-salt (HS) seawater containing twice the normal molarity of NaCl (sodium chloride) was applied, a procedure previously reported to completely deciliate the ectodermal surface of the embryo [43]. Embryos between early gastrula and prism stage were treated with HS seawater for 60–90 s and returned to regular seawater until untreated control embryos reached late gastrula to pluteus stages. Embryos were fixed, assessed for developmental defects and/or processed for IF, SEM or *in situ* hybridization. First we analyzed whether this procedure was suited to remove archenteron cilia. Swimming behavior was instantly impaired, as described for Ciliobrevin above (not shown). Embryos were fixed 20 min after treatment and subjected to IF analysis of cilia or SEM analysis. While normal ciliation was seen in SEM photographs of untreated embryos, HS-treated specimens were devoid of cilia, both on the outer surface and at the proximal (posterior) end of the archenteron cavity (Fig. 3a, b). To investigate whether cilia were removed along the entire archenteron, we analyzed optical sections of treated embryos using IF. Control embryos displayed normal ciliation along the extended archenteron. In contrast, about half of the HS-treated embryos lacked cilia altogether, with the remainder of specimens displaying a small number of very short cilia remnants (Fig. 3c, d; Additional file 1: Figure S1i). These experiments demonstrated that HS treatment was an efficient tool to remove archenteron cilia from the embryo.

Because of the proven function of cilia in signal transduction in different species [44, 45], we asked whether deciliation affected normal embryonic development. The protocol applied here has been previously used to remove ectodermal cilia at different stages of development, without any reports of developmental defects [46–49]. Stephens et al. (1977) even applied multiple rounds of deciliation (up to ten times) which resulted in phenotypically normal pluteus larvae [50]. In order to confirm that the HS-protocol did not interfere with normal embryogenesis in our experiments as well, >1.500 embryos were deciliated in 11 independent experiments at different time points during development, between late blastula and late gastrula stages. Treated specimens were fixed and assessed for developmental delays or phenotypic alterations at time points when untreated control embryos had reached a) late gastrula with fully elongated archenteron; or b) early pluteus stages. In both sets of experiments, no phenotypical difference was noted between control and HS-treated samples (Additional file 1: Figure S1e–h). Quantitative evaluation of experiments revealed no temporal delay in either of these groups, besides minor and random fluctuations that were sometimes observed (Additional file 1: Figure S1j). Together with the previously published deciliation approaches, these control experiments demonstrate that the protocol applied here efficiently removed archenteron cilia without impairment of normal development.

In a final series of experiments, we tested whether deciliation impacted on *nodal* asymmetry in the archenteron and ectoderm, which we hypothesized based on our descriptive analysis of archenteron cilia. HS treated embryos indeed revealed grossly altered patterns of *nodal* expression at late gastrula stages, when control embryos displayed unilateral patterns in both, the archenteron and the ectoderm (Fig. 3e, f). Surprisingly, HS treatment at early to mid-gastrula stages, i.e. just before asymmetric induction of the *nodal* cascade, resulted in an expanded, bilateral ectodermal expression of *nodal* in the vast majority of treated specimens. The same result was obtained with *pitx2* (Fig. 3g, h), a direct target of Nodal [51]. Bilateral activation of the Nodal cascade was especially evident when analyzed in the developing coelomic pouches of early pluteus stage embryos, using *pitx2* as a late LR marker gene (Fig. 3j, k). The archenteron domain of *nodal* in late gastrula stage embryos, however, was mostly undetectable (Fig. 3e, f).

Next we tested whether high-salt treatments resulted in a stress-induced activation of MAPK/p38-mediated ectopic activation of *nodal* [52]. If it were, inhibition of MAPK/p38 should rescue the deciliation-induced aberrant activation of the Nodal cascade. Blastula-stage embryos were treated with the MAPK/p38 pathway-specific inhibitor SB203580 [52], to test the efficiency of the drug, which resulted in dorso-ventral axis defects (Additional file 1: Figure S1k–n). When embryos were treated after deciliation at mid to late gastrula stages, development was not impaired but specimens still exhibited bilateral *pitx2* expression in the coelomic pouches at early pluteus stage (Fig. 3l–o). These experiments thus demonstrated that the expanded expression pattern of LR marker genes after deciliation was not due to MAPK/p38-mediated over-activation of Nodal signaling during gastrulation.

In order to determine whether there was a sensitive time period, i.e. whether cilia were required only during certain developmental stages, we performed a time course of deciliation and treated specimens with high salt at defined points of development: early gastrula, mid gastrula, late gastrula or prism stage. Based on cilia-driven symmetry breakage in the vertebrates, the time window was expected to be rather narrow. Figure 3i demonstrates that deciliation at very early to mid-gastrula stages, before full extension of the archenteron and before asymmetric *nodal* expression, caused aberrant LR development. Embryos treated after this point, during late gastrula to early prism stages, showed normal unilateral expression of *nodal*, i.e. the sensitive time window closes during late gastrulation.

In summary, our work demonstrates that the sea urchin archenteron harbors polarized and motile monocilia, which are required for LR axis determination during gastrulation.

## Discussion

**Fig. 4:**
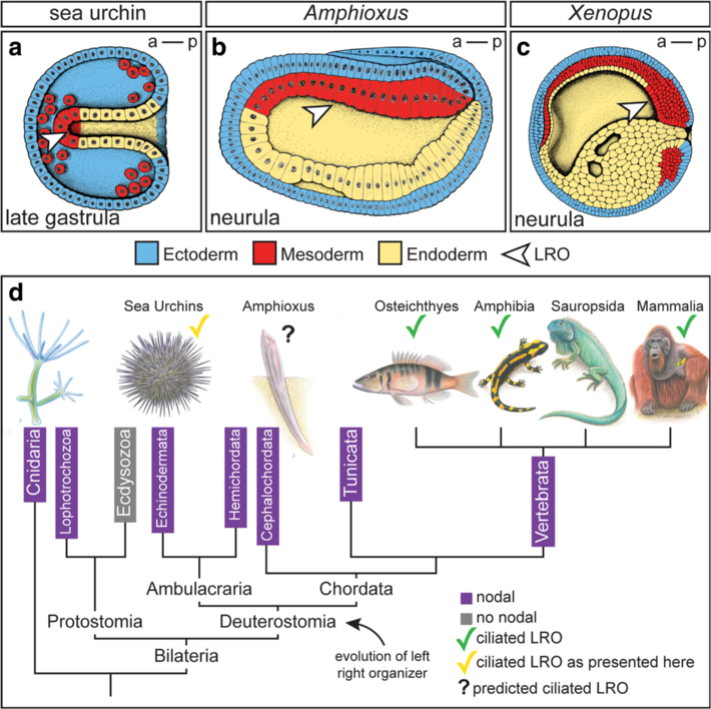
The ciliated Left-Right Organizer: a synapomorphy of the deuterostomes. **a**–**c** Schematic drawings of sea urchin (**a**), amphioxus (**b**) and *Xenopus* (**c**) gastrula/neurula stage embryos (anterior to the left). In all cases the archenteron harbors cells of mesodermal fate (SM, red), which in frog and sea urchin are ciliated and function as LROs. The position of mesodermal LROs is indicated by an arrowhead. **d** *nodal* is evolutionary conserved among metazoans except for the ecdysozoa. Deuterostomes share a ciliated LRO as a common synapomorphy. Drawings adapted in parts from Blum et al. 2014 [3]

Theoretical considerations and deductive logics have previously led us to propose that sea urchin embryos possess an ancestral LRO, homologous to that of the vertebrates [11]. Here we demonstrate in two species that sea urchin gastrula embryos indeed display a mesendodermal monociliated archenteron, which is reminiscent of the vertebrate LRO (Fig. 4a). Importantly, cilia were required for asymmetric *nodal* induction, arguing for a conserved cilia-based mechanism for LR symmetry breakage in the deuterostome lineage.

Common features between the sea urchin archenteron and vertebrate LROs include polarized and motile monocilia as well as expression of *dnah9* and *foxj1*, two genes which are mandatory for generation of leftward flow. Although we were not able to visualize a fluid flow within the archenteron directly, we propose that cilia-dependent symmetry breakage is a conserved feature between sea urchin and the vertebrates. In order to qualify as the *nodal* inducing event, cilia-driven symmetry breakage should occur at the right time, before asymmetric *nodal* induction, and in the right place, i.e. at the archenteron. Besides the presence of motile cilia itself, the expression of vertebrate LRO components *dnah9*, *foxj1,* and *pkd2* in this very region are in perfect agreement with this proposal (Fig. 2).

Not every LRO feature seems to be conserved between sea urchins and the vertebrates, though. While flow-perceiving (and *nodal* expressing) cells in vertebrates are located at the posterior pole of the embryo, close to the blastopore or proximal end of the gut, these cells reside at the anterior tip of the archenteron in the sea urchin late gastrula embryo, at its distal end, as deduced from the *nodal* expression domain (Fig. 3e and [37, 53]). Interestingly, an intermediate scenario is encountered in amphioxus. Here, like in amphibians, a bilateral *nodal* expression domain was found, but in the anterior part of the archenteron like in sea urchins. The expression on the right side of amphioxus later disappears, possibly due to a leftward cilia-driven flow [3, 26] (Fig. 4b). This reasoning is further supported by the recent description of a *dand5* homolog, which was found co-expressed with *nodal* but down-regulated on the left side, resembling the situation at the vertebrate LRO [28]. Interestingly, a *dand5* orthologue was not reported for *P. lividus*, nor was it found in *S. purpuratus* [53]. Considering just two features, localization of the supposed LRO and presence or absence of *dand5*, sea urchins, cephalochordates and vertebrates could represent three distinct states, indicative of evolutionary transitions: while sea urchins and amphioxus share an anterior (distal) position of the LRO within the archenteron, amphioxus and vertebrates both possess an orthologue of the Nodal antagonist *dand5*, but only the vertebrates localize their LRO close to the blastopore. What kind of functional adaptations underlie these variations remains to be investigated.

There are additional LR components worth being considered in the evolutionary context, namely *pkd2* and *bicc1*, both of which were expressed in early sea urchin gastrula mesendodermal tissues during invagination (Fig. 2i–k, Additional file 1: Figure S1 and [41]). *bicc1* in mouse and frog functions to polarize LR cilia [40]. In that sense the expression in sea urchins correlated with that in frog and mouse: it is precisely the *bicc1* expressing cells which harbor cilia polarized to the posterior pole in mid gastrula stage embryos in the sea urchin, and which we here report to be motile (cf. Fig. 1h and Additional file 1: Figure S1 and Additional file 2: Movie 1). Expression differed in another aspect, however, as *bicc1* mRNA was not colocalized with *nodal* expression at the archenteron tip, different from the vertebrates, where *bicc1* is co-expressed with *nodal* in the LRO-flanking cells - and might be involved in sensing of flow [40]. It would be interesting to know where *bicc1* is expressed in amphioxus, around the blastopore or in the anterior mesoderm.

*pkd2* in the sea urchin embryo was expressed in the vegetal mesoderm and endoderm at early to mid-gastrula stages and at the tip of the archenteron. This expression is remarkable, as *pkd2* has not been reported to be transcriptionally up-regulated in or at any vertebrate LRO. The encoded protein, the calcium channel Polycystin-2, however, is present in mouse and fish LROs*,* where it is involved in flow sensing [19, 20, 22]. Functional conservation of symmetry breakage is further supported by the expression of *pkd2* in *Xenopus* gastrula embryos, where it is highly expressed in the mesendodermal ring around the blastopore, reminiscent of the vegetal mesoderm expression in sea urchins (our unpublished observations). Again, expression in amphioxus has not been reported as yet but should be highly informative. Taken together, the presence of cilia and the expression of conserved LR genes strongly argue for a conserved role of motile archenteron cilia in LR symmetry breakage.

The most convincing result, however, is presented by our analysis of cilia function, where deciliation at a time point just prior to asymmetric induction of *nodal* in the archenteron resulted in altered expression of the Nodal cascade. Molecular asymmetries were lost, with a differential response of *nodal* and *pitx2*. While archenteron *nodal* was mostly absent or below the detection level of the experiment, the ectodermal domain and later on *pitx2* in the coelomic pouches became expanded and bilateral (Fig. 3). These observations cannot be attributed to the absence of archenteron Nodal. When Nodal was experimentally manipulated in the archenteron to be absent or expressed in a bilateral manner, the ectodermal domain remained asymmetrical, although in a randomized manner, being activated either on the left or on the right side [33, 37]. The ectoderm is still competent to express *nodal* on both sides during gastrulation, as shown by Activin treatment of early gastrula embryos [33]. The maintenance of unilateral ectodermal *nodal* expression might thus be explained by an additional ectodermal cilia function, which was ablated in high salt-treated embryos as well, but we can only speculate on this issue. Our results would be explained if external cilia had a function in restricting Nodal cascade activation unilaterally in the ectoderm, and the archenteron cilia provided a biasing cue, as they do during vertebrate LR axis determination [2].

While this manuscript was under review, two papers were published that dealt with symmetry breaking in sea urchin embryos. In agreement with our conclusions, Takemoto et al. deduced that cilia were required for symmetry breakage in sea urchins. Application of an inhibitor of motile cilia to early blastula stage embryos resulted in a loss of *nodal* asymmetry, archenteron cilia, however, were not analyzed in this study [54]. Warner et al. (2016) injected 1-cell stage embryos with a kinesin-2 antibody, which removed all cilia from the time point of injection onwards. *Nodal* was strongly reduced in the four embryos analyzed, while asymmetric *SoxE* expression in the coelomic pouches was retained in the six embryos included in this one experiment. The authors concluded that cilia were not required for symmetry breakage but rather for hedgehog signaling-mediated *nodal* maintenance [55]. We strongly disagree with this conclusion for the following reasons: (1) Lepage and colleagues have shown that asymmetric *SoxE* expression occurs independent of archenteron Nodal; (2) the removal of all cilia from the earliest developmental stages onwards potentially impacts on many more signaling pathways, such as for example hedgehog, as shown by Warner et al (2016) [55], and thus most probably impact on stages before archenteron cilia emerge; (3) attenuated *nodal* expression by permanent cilia ablation, as described by Warner et al., might be the result of a) loss of archenteron cilia function, which causes bilateral expanded *nodal* expression as shown in our present work, followed in time by b) loss of hedgehog-mediated *nodal* maintenance, as described by Warner et al. (2016).

We like to extend our evolutionary considerations to the precursor tissue of the LRO in the sea urchin. Keller and colleagues previously revitalized the concept of the superficial mesoderm (SM) in the chordate lineage. Best characterized in amphibians, the SM of the pre-gastrula embryo gives rise to the archenteron LRO during gastrula/neurula stages to end up in the axial and paraxial mesoderm during later embryogenesis [8]. Besides the birds, which lack an LRO, the SM has been identified in most chordate lineages [8, 11, 56] (Fig. 4a–d). In the sea urchin mesenchyme blastula, the non-skeletogenic mesoderm, together with the endoderm, locates superficially at the vegetal pole as well, reminiscent of the situation in amphibians [8, 57]. Of the known SM marker genes in the frog, *foxj1* [58–60], *nodal3* [58] and *wnt11b* [61], only *foxj1* has been analyzed in sea urchins (Fig. 2e–g). Its mRNA localization in the early gastrula vegetal cells indeed argues for a conserved blastula stage SM (Fig. 2h). Furthermore, like in vertebrate SM tissues, these cells show accumulation of nuclear ß-catenin at the very time point when the SM is specified [62]. This is of relevance, as *foxj1* expression has been shown to be directly induced by canonical Wnt signaling.

## Conclusions

We conclude that the early sea urchin embryo represents an ancestral deuterostome state with a vegetal SM at blastula stages, which transforms into an archenteron LRO, where motile cilia are necessary to break the bilateral symmetry. Cilia-driven symmetry breakage thus should represent a synapomorphy of the deuterostome lineage.

## Methods

### Animals and embryo manipulation

Adult *P. lividus* were collected in Pula/Croatia in early summer during the natural breeding season and reared short-term in the laboratory. Adult *S. pallidus* were collected at the MSU White Sea Biological Station, Kandalaksha Bay/Russia during the summer. Embryos were obtained by artificial fertilization after injecting adult specimen with 1 ml of 0.5 molar KCl into the oral field to obtain sperm and oocytes and raised in artificial sea water (ASW) containing antibiotics at 18–20 °C or 4 °C for *P. lividus* and *S. pallidus*, respectively.

### High-salt and Ciliobrevin D treatment

Early or mid-gastrula stage embryos were transferred to 3 % ASW, which contained high concentrations of NaCl (1 M) but regular seawater molarities of all other ions, and kept for 60–90 s before washing and transferring to regular ASW. Efficiency of deciliation was controlled in the microscope by absence of embryonic swimming behavior. Subsequently, embryos were incubated until the desired stages for treatment or analysis were reached. For dynein motor inhibition, embryos were transferred to ASW containing 50 μM Ciliobrevin D at early gastrula stage and incubated until control untreated specimens reached late gastrula stage. For SB203580 treatment (ENZO Life Sciences), embryos were transferred in sterile sea water containing either 20 μM or 5 μM of SB203580 or DMSO as a control in the same dilution and grown until pluteus stage.

### RT-PCR

Total RNA was isolated from embryos at various stages of development using peqGOLD Trifast (VWR International GmbH, Erlangen/Germany) and cDNAs were prepared using standard protocols. Primers used for PCR amplification of fragments were designed using ESTs:

plbicc1_fwd: 5′gcctgaggtttggttagtgc3′, plbicc1_rev: 5′cgatctgtcctgcaatagaaacc3′, plfoxj1_fwd: 5′gtcaacacattccaaccatctc3′, plfoxj1_rev: 5′ctcttctttggcatggtctg3′, pldnah9_fwd: 5′tgccaactttcaatcatatttca3′, pldnah9_rev: 5′atgaacactctacatcagagatc3′, plpkd2_fwd: 5′atccctctggagaacgagac3′, plpkd2_rev: 5′gcaaacatgacagtgaatcctc3′, plnodal_fwd: 5′tttcttcgctccattcctcc3′, plnodal_rev: 5′gaactaagacggctccttcc3′

### Cloning of constructs

Partial coding sequences of *plbicc1, plfoxj1, pldnah9, plpkd2 and plnodal* were cloned in the pGEM-T Easy vector system and verified by sequencing.

### RNA *in situ* hybridization

Embryos were fixed in paraformaldehyde (PFA) 4 % PFA for 30 min, stored in 100 % Ethanol at -20 °C and processed following standard protocols. Digoxigenin-labeled RNA (Roche) probes were prepared with SP6 or T7 RNA polymerase on linearized pGEM-T Easy templates (Promega). *In situ* hybridization was modified from [63]. Initial rehydration steps were performed with 1 % BSA in PBS to avoid agglutination of embryos. All steps were performed either in glass wells or custom made plastic tubes with meshwork.

### Immunofluorescence

Embryos were fixed in 4 % PFA in ASW for 30 min, washed with PBS, permeabilized with PBS/0.1 % Triton X-100 (Sigma), blocked with CAS-Block™ Histochemical Reagent (Life technologies) and incubated with primary antibodies overnight at 4 °C. Secondary antibodies were incubated for 2 h at room temperature. Primary antibodies used in this study were: anti-acetylated α-tubulin (Sigma, T6793); anti-α-tubulin (Sigma, T6199). Reagents used for visualization of nuclei included DAPI, Hoechst and propidium iodide. To visualize cell membranes, Phalloidin-Tetramethylrhodamin B isothiocyanate (Sigma, P1951) or Alexa 488 Phalloidin (Life technologies, A12379) were used.

### Scanning electron microscopy

Wild-type or HS-treated embryos were fixed in 4 % PFA containing 0.2 % Glutaraldehyde in PBS. Preparation of embryos for SEM followed standard protocols. Prior to sputter-coating with gold, some embryos were manually broken using a pipette tip, in order to visualize the archenteron surface.

### High-speed videography of cilia motility in gastrula stage embryos

To observe ciliary motility directly, gastrula stage embryos were either transferred into a solution containing 0.5–1.0 % methylcellulose (Sigma) in ASW in order to slow down beating of the cilia in the forming archenteron cavity (for early to mid-gastrula stages), or positioned within a nitex screen (SEFAR, Germany) to focus on the central archenteron cavity (mid-late gastrula stages). Imaging was performed using a Hamamatsu ORCA-Flash 4.0 Digital CMOS camera mounted on a Zeiss Imager.M1 microscope equipped with a Plan-Apochromat 100x/1.4 oil objective. Acquisition of frames was performed using the Zeiss ZEN software. Fiji [64] was used for temporal difference imaging, i.e. for visualization of movements against a ‘static’ background. Each frame of a time-series was subtracted from its consecutive frame (t_n_-t_n+1_), resulting in different pixel grey values with black indicating no change in between two frames.

### Photo-documentation and picture analysis

IF pictures were taken on a Zeiss Observer. Z1/LSM 700 equipped with a 63x objective (C-Apochromat 63x/1.2 W Corr). Photographs of embryos after *in situ* hybridization were taken on an Axioskop 2 mot plus (Zeiss, Germany) and processed in Adobe Photoshop. Figures were assembled using Adobe Illustrator.

## Additional files

Additional file 1: Figure S1. mRNA expression of *bicc1* (a–d) and overall normal development of *Paracentrotus lividus* embryos upon high salt induced deciliation (e–j) or upon early MAPK/p38 inhibition (k–n). (PDF 877 kb) Additional file 2: Movie 1. Archenteron cilia are motile. Real-time movie of early to mid gastrula stage embryo in lateral-vegetal view reveals fast movement of polarized cilia on laterally invaginating cells at the posterior aspect of the archenteron (cf. Fig. 1h). Bright-field movie (a) and high-magnification (white box) of the inner archenteron wall (a’). (b) Temporal difference imaging rendered version of the same movie, which highlights pixel grey value differences between two consecutive frames. Movie was acquired at 55fps (cf. methods for details). (AVI 8410 kb) Additional file 3: Movie 2. Monocilia inside the archenteron of gastrula stage embryos rotate Real-time movie of mid to late gastrula stage embryo in vegetal view, focusing on the central archenteron region, reveals fast rotating monocilia inside the archenteron (cf. Fig. 1g). (a) bright-field movie. (b) Temporal difference imaging rendered version of the same movie, which highlights pixel grey value differences between two consecutive frames. (a’, b’) High-magnification area as outlined in (a, b). Movie was acquired at 35fps. Please note the rotating cilia on the inner epithelium of this embryo. (AVI 7326 kb)
