# Supplementary material for: Cilia are required for asymmetric nodal induction in the sea urchin embryo
Source: BMC Dev Biol. 2016 Aug 23;16(1):28. doi: 10.1186/s12861-016-0128-7 (PMC4994401; doi:10.1186/s12861-016-0128-7)
Supplement: Additional file 1: Figure S1. — mRNA expression of bicc1 (a–d) and overall normal development of Paracentrotus lividus embryos upon high salt induced deciliation (e–j) or upon early MAPK/p38 inhibition (k–n). (PDF 877 kb) [file 12861_2016_128_MOESM1_ESM.pdf]

Supplementary Information

Cilia are required for asymmetric *nodal* induction in the sea urchin embryo

Matthias Tisler[1], Franziska Wetzel[1#], Sabrina Mantino[1#], Stanislav Kremnyov[2], Thomas Thumberger[1+], Axel Schweickert[1], Martin Blum[1], Philipp Vick[1*]

Supplementary Figures

Supplementary Figure 1

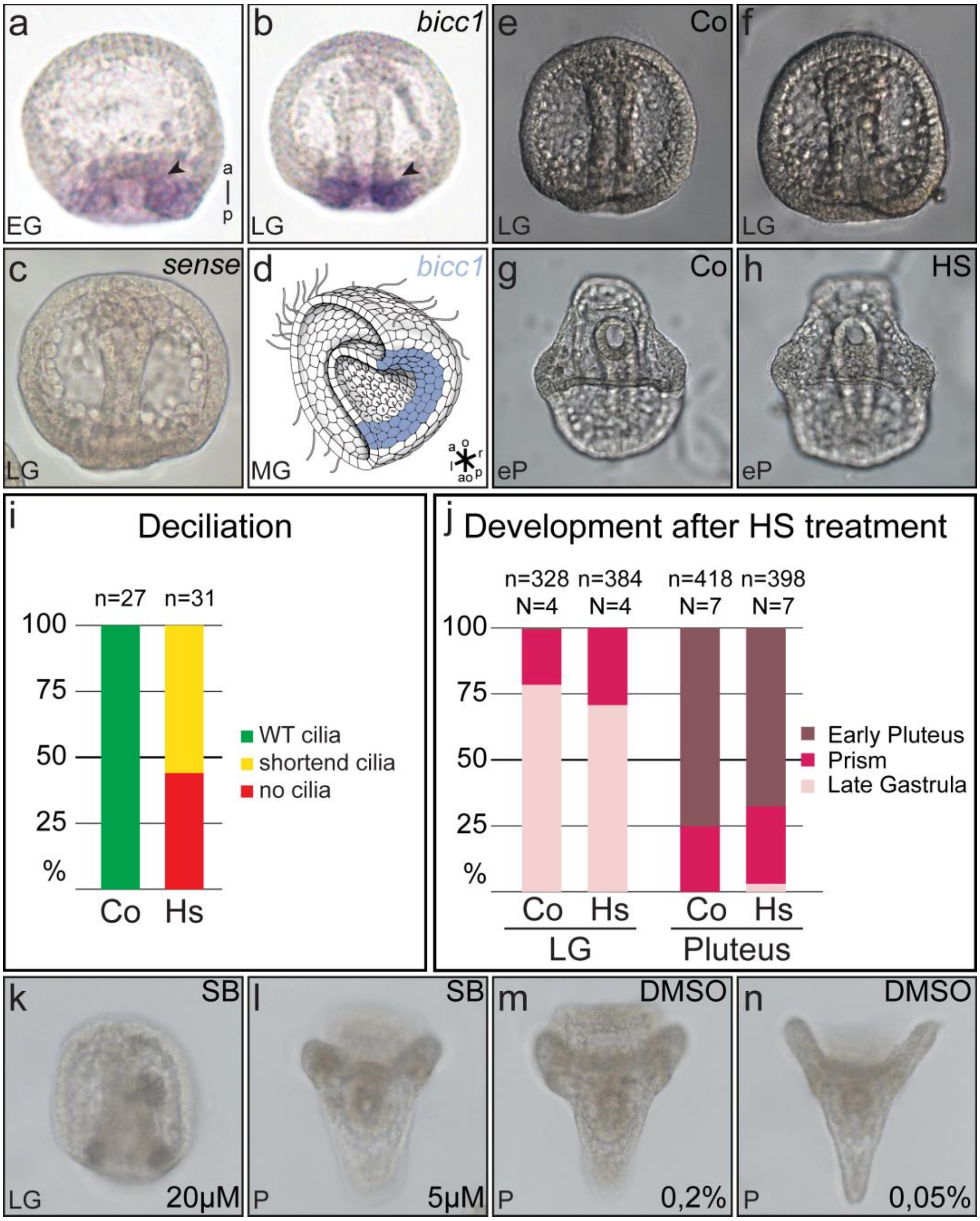

mRNA expression of *bicc1* (a-d) and overall normal development of *Paracentrotus lividus* embryos upon high salt induced deciliation (e-j) or upon early MAPK/p38 inhibition (k-n)

(a-d) *bicc1* transcripts were detected in the invaginating mesendodermal cells during gastrulation. (c) A control embryo stained with sense RNA showed no staining. Embryos deciliated between late blastula to late gastrula stages developed without apparent phenotype to late gastrula (f) or early pluteus stage (h), respectively, as compared to control embryos (e, g). (i) Quantification of deciliation efficiency after high salt treatment, based on assessment of IF using an antibody against acetylated

tubulin (n = number of embryos analyzed). (j) Developmental stages of wild-type and high-salt treated embryos. Summary of results from a total of 11 deciliation experiments performed at early (EG), mid (MG) or late gastrula (LG) stages and scored when most controls developed to late gastrula stage (left bars) or early pluteus stage (right bars), respectively (n = number of embryos; N = number of experiments). Please note that there was no overall delay in development after HS treatment. (k-n) Embryos after MAPK/p38 inhibition during blastula showed dose-dependent DV patterning phenotypes resembling late gastrula stages (LG) at early pluteus (P) stages (k, l) while DMSO control embryos developed normally (m, n). Drawings in (d) adapted from Blum et al. 2014[1].

Supplementary References

01. Blum, M., Feistel, K., Thumberger, T. & Schweickert, A. The evolution and conservation of left-right patterning mechanisms. *Development* **141,** 1603–1613 (2014).
